# Metal on metal hip resurfacing versus uncemented custom total hip replacement - early results

**DOI:** 10.1186/1749-799X-5-8

**Published:** 2010-02-18

**Authors:** Nemandra A Sandiford, Sarah K Muirhead-Allwood, John A Skinner, Jia Hua

**Affiliations:** 1 The London Hip Unit, 4th Floor, 30 Devonshire Street, London, UK, W1G 6PU; 2 The Royal National Orthopaedic Hospital, Stanmore, Middlesex, UK, HA7 4LP

## Abstract

**Introduction:**

There is no current consensus on the most appropriate prosthesis for treating symptomatic osteoarthritis (OA) of the hip in young, active patients. Modern metal on metal hip resurfacing arthroplasty (HR) has gained popularity as it is theoretically more stable, bone conserving and easier to revise than total hip arthroplasty. Early results of metal on metal resurfacing have been encouraging. We have compared two well matched cohorts of patients with regard to function, pain relief and patient satisfaction.

**Methods:**

This prospective study compares 2 cohorts of young, active patients treated with hip resurfacing (137 patients, 141 hips) and custom uncemented (CADCAM) stems (134 patients, 141 hips). All procedures were performed by a single surgeon. Outcome measures included Oxford, WOMAC and Harris hip scores as well as an activity score. Statistical analysis was performed using the unpaired student's t-test.

**Results:**

One hundred and thirty four and 137 patients were included in the hip replacement and resurfacing groups respectively. The mean age of these patients was 54.6 years. The mean duration of follow up for the hip resurfacing group was 19.2 months compared to 13.4 months for the total hip replacement group.

Pre operative oxford, Harris and WOMAC scores in the THA group were 41.1, 46.4 and 50.9 respectively while the post operative scores were 14.8, 95.8 and 5.0. In the HR group, pre- operative scores were 37.0, 54.1 and 45.9 respectively compared to 15.0, 96.8 and 6.1 post operatively. The degree of improvement was similar in both groups.

**Conclusion:**

There was no significant clinical difference between the patients treated with hip resurfacing and total hip arthroplasty in the short term.

## Introduction

Traditionally the primary indication for total hip arthroplasty (THA) has been incapacitating pain which could not be sufficiently relieved by conservative means and for whom the only surgical alternative was excision of the hip joint (Girdlestone resection arthroplasty). At that time post-operative function was secondary to pain relief [1].

Pain remains the primary indication for surgery and not limitation of motion, leg length inequality or radiographic features. With modern advances in implant design and the development of new bearing surfaces, improved implant survival has been demonstrated [2-12]. This has led to younger and more active patients requesting hip arthroplasty. This young, active population also has a desire to maintain an active lifestyle [8,12-19] and remain the biggest challenge for arthroplasty surgeons[20].

Our policy has been to use uncemented THA with computer-aided-design computer-aided-manufacture (CAD CAM) stems in young, active patients since 1991, most recently utilising ceramic-on-ceramic bearing surfaces. Since August 2000, hip resurfacing became an option and the Birmingham Hip Resurfacing prosthesis consisting of chromium cobalt metal-on-metal bearing surfaces (BHR, Smith and Nephew, Warwick, UK)[10] has been used where appropriate in this patient group. Its use as an option available to young patients on the National Health Service (NHS) was approved by The National Institute of Health and Clinical Excellence (NICE) in the United Kingdom in June 2002.

It has been postulated that the functional outcomes of hip resurfacing (HR) exceed those of THA [9-11,19,21].

### Aim

The aim of this study is to assess whether young, active patients treated with hip resurfacing arthroplasty have better functional and symptomatic outcomes when compared to those treated with custom computer aided design computer aided manufacture (CADCAM) uncemented THA prostheses in the early post operative period.

### Patients and Methods

This study was performed between August 2000 and November 2002. All patients included in the study had a primary diagnosis of osteoarthritis of the hip and were under 65 years of age at the time of their operation. Other than minor dysplasia all hips were anatomically normal. Patients with Crowe types 3 and 4 developmental dysplasia of the hip (DDH) were excluded. All procedures were performed by the senior author (SM-A). Two patients had one of each of the above procedures on contra-lateral sides.

Initially there was a reluctance to use this prosthesis in female patients over 50 years old due to the relatively increased rate of osteopaenia in this age group. The use of HR prostheses increased as the learning curve progressed. This meant that patients were not randomised and HR prostheses were used more towards the latter part of the study. This accounted for the discrepancy in the duration of follow up between the two groups. The criteria for inclusion into the study was similar in both groups ie. debilitating hip osteoarthritis in patients under 65 years of age. Total hip arthroplasty was offered if there was radiological evidence osteopaenia or segmental collapse of the femoral head.

### Hip Resurfacing (HR) Group

One hundred and thirty seven consecutive patients (141 hips) who had hip resurfacing procedures were included in this study. This series included 93 males and 44 females. Their mean age was 55.3 years (28.4-64.6 years).

### Total Hip Arthroplasty (THA) Group

One hundred and thirty four consecutive patients (75 males, 59 females) were included in this group. Their mean age was 53.9 years (Range 24.8-64.6 years).

### Clinical Outcomes

Patients were followed up at 1 month, 3 months, 6 months and yearly post operatively. At each visit they were interviewed, examined and assessment of hip scores were performed. Clinical outcomes were assessed by the Oxford, Western Ontario Macmasters (WOMAC) and Harris hip scores all of which are validated [20-24]. These scoring tools were used both pre and post-operatively. Activity level was measured using the modified (University of California Los Angeles) UCLA activity score [25].

The hip scores all evaluated degree of symptoms ie severity of pain, night pain and the degree of functional deficit ie effect on walking distance, self caring activities and other activities of daily living eg stair climbing as well as clinical parameters eg hip flexion in order to arrive at a final score. In the case of the WOMAC and Oxford scores a low score indicates good function while the opposite is true for the Harris Hip Score. The UCLA score assigns a numerical value to the level of function of the patients.

Differences between the results of the 2 groups were evaluated by using an unpaired student's t-test.

## Results

**Figure 1:**
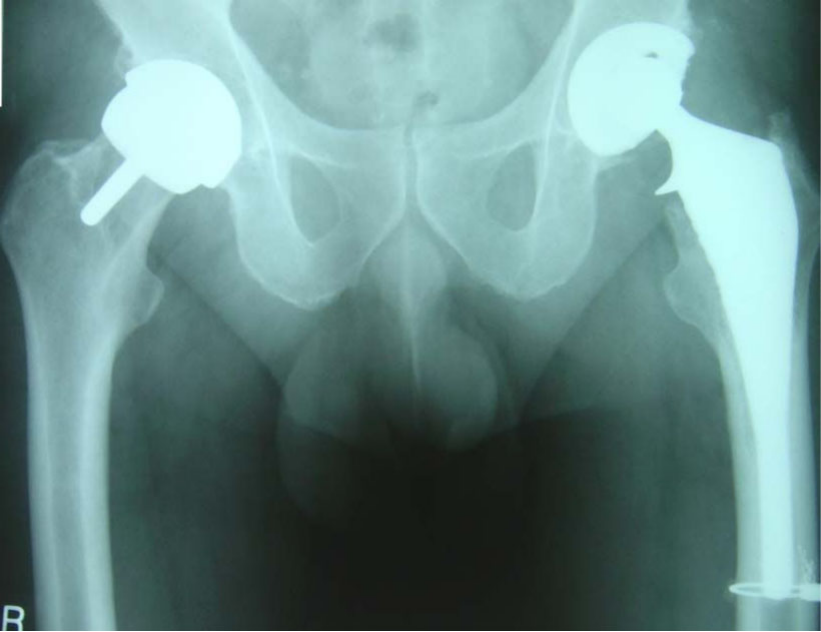
**Patient with bilateral procedures**. Right-Birmingham Hip Resurfacing arthroplasty. Left-CADCAM primary total hip replacement.

Between August 2000 and November 2002 141 HRs and 141 THAs were performed. Two patients had one of each of the above procedures on contra-lateral sides (Figure 1). While one of these patients thought both hips functioned equally well, the other complained of occasional discomfort in the scar of the THA therefore she favoured the resurfacing side. The THA was performed via a minimally invasive posterior approach (incision length 8.8 cm) while the HR was performed via a 15 cm incision (posterior approach). There was no functional difference or difference between the hip scores on either side. Patient demographics are summarised in Table 1.

**Table 1:** Demographics of our patient cohorts

Patient Demographics	THA^1^	HR^2^
No of pts in study	134	137
Males	75	93
Females	59	44
UCLA Score (pre-op)	2	9
UCLA Score (post-op)	3	9
Mean age (range)	53.9 (24.8 - 64.6)	55.3 (28.4-64.6)
Mean BMI^3^	26.0 (17.2 - 37.6)	26.0 (18.2 - 36.1)

^1^Total Hip Arthroplasty

^2^Hip Resurfacing

^3^Body Mass Index

### Total Hip Arthroplasty (THA) (Figure 2)

**Figure 2:**
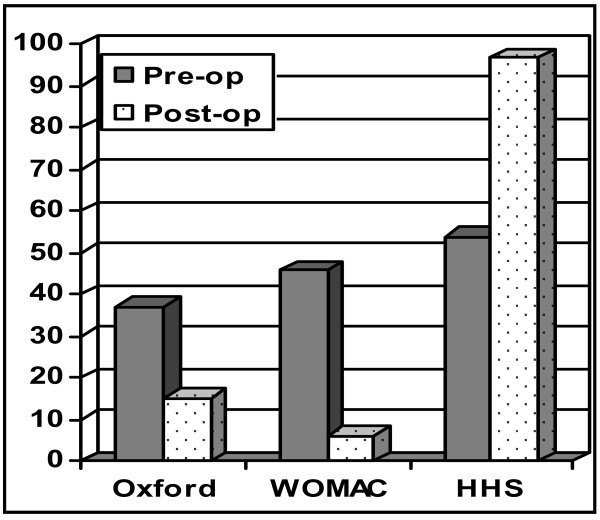
**Pre and Post operative scores in the total hip arthroplasty (THA) group**.

Two patients died of unrelated causes since operation. One patient refused to participate in the study. 3 patients did not respond to the questionnaires by mail or telephone. This left 134 out of 137 patients (97.1%) in the study group (75 males and 59 females) with an average follow-up of 19.2 months (3.0 - 38). Eighty per cent (107 patients) were reviewed at a minimum of 24 months.

The average pre-operative Harris, Oxford and WOMAC scores were 46.4 (7 - 87), 41.1 (range 16 - 75) and 50.9 (3 - 96) respectively. Average post operative scores were 95.8 (65 - 100), 14.8 (12 - 33) and 5.0 (0 - 39) respectively.

The Harris Hip Score increased by 49.4 points, an improvement of 49.4%. The Oxford Hip score improved by 26.3 points, an improvement of 54.8% while the WOMAC score improved by 45.9 points, correlating to a 47.8% improvement in function. There were no failures requiring revision

### Hip Resurfacing (HR) (Figure 3)

**Figure 3:**
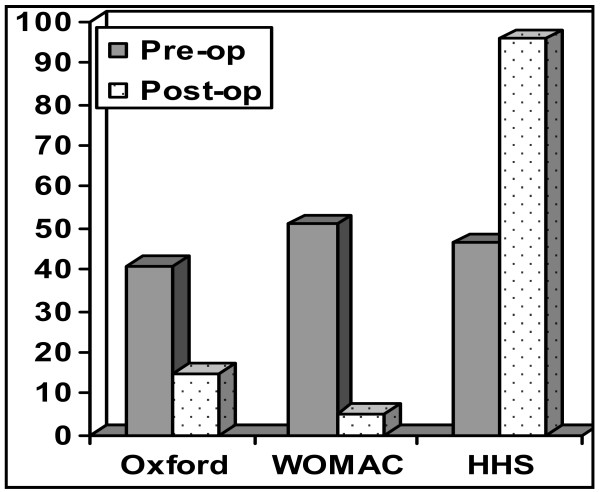
**Pre and Post operative scores in the hip resurfacing (HR) group**.

One patient died of an unrelated cause since operation. Two patients did not respond to the questionnaires by mail or telephone. This left 137 out of 139 patients (response rate = 98.6%) in the study group (93 males and 44 females) with an average follow-up of 13.4 months (Range 3 - 36.7 months). Eighty nine patients (60%) were reviewed at a minimum of 24 months.

The mean pre-operative Harris, Oxford and WOMAC hip scores were 54.1 (7 - 97), 37.0 (13 - 57) and 45.9 (1 - 94) respectively. Mean post operative scores were 96.8 (59 - 100), 15.0 (12 - 35) and 6.1 (0 - 56) respectively.

The mean Harris hip Score improved by 42.7 points (42.7%), while the Oxford score improved by 22.0 points to 39.8 points, representing a 41.5% improvement in function postoperatively.

There were no failures requiring revision.

There were no statistically significant differences in the post-operative scores within either group (p values: Oxford = 0.60, WOMAC = 0.31, Harris = 0.15). The THA group had worse preoperative function (p values: Oxford = 0.0007, WOMAC = 0.0323, Harris = 0.0005).

The percentage improvement between pre-operative and post-operative responses was significantly better in the THA group than the HR group (p values: Oxford = 0.0001, WOMAC = 0.0136, Harris = 0.0028).

### Activity Scores

These patients were young, motivated individuals who played at least one sport two or more times weekly. Each patient played an average of 3 activities (range 2-6). Activity levels were measured using a modification of the University of California Los Angeles (UCLA) Activity Level Scale (Appendix 1). Mean preoperative level was 3 compared to 9 postoperatively. Patients were advised against some of these sports, including skiing but they participated regardless. The level of activity achieved along with relief of pain contributed to our patients' satisfaction with the procedure.

### Complications (Table 2)

**Table 2:** Complications noted in each patient group

	THA	HR
Dislocation	2	0
DVT	0	3
Infection	2	2
Revisions	0	0
Deaths 3		

In the Hip Resurfacing (HR) group there were 3 superficial wound infections and 2 cases of deep vein thromboses (DVT's). The infections all occurred within 30 days postoperatively and were treated with oral antibiotics. The DVT's occurred within this time period as well and were confirmed by duplex Doppler imaging. Neither case progressed to pulmonary emboli. There were no dislocations. One patient died of causes unrelated to her surgical procedure.

In the THA group there were 2 dislocations and 2 cases of superficial wound infections. The dislocations were managed by closed reduction. No abduction braces were used. There were no cases of recurrent dislocations. The cases of superficial infection resolved with oral antibiotics. There were no DVT's in this group. Two patients had died from unrelated causes at the time of last follow up.

## Discussion

The overall success of total hip arthroplasty has not been reflected in young, active patients. As a result the majority of contemporary research has been focused towards improving results particularly in the younger, more active patient demanding a high functional outcome. Total hip arthroplasty has previously been avoided in this group due to concerns of durability of prostheses and projected need for multiple revision procedures with progressive loss of bone stock.

Hip resurfacing has become more popular in this group following advances in engineering and metallurgy. Modern metal-on-metal bearings appear to offer excellent wear properties when compared to historical resurfacing designs, which were mainly metal-on-polyethylene [2,3,10,26]. HR seems to be an attractive concept which offers durable bearing surfaces with low wear, bone conservation and simple revision options-particularly on the femoral side.

The results obtained when comparing different groups of patients can be confounded by the presence of multiple variables. The groups of patients presented are well matched for several reasons. They represent a single surgeon series in which the same surgical approach (posterior approach) and method of closure (capsular and short external rotator repair) were used. Perioperative management was similar between the two groups as well. All patients were mobilised from day 1 post operatively by physiotherapists. They progressed from crutches to sticks and were discharged once they were safe on these.

**Figure 4:**
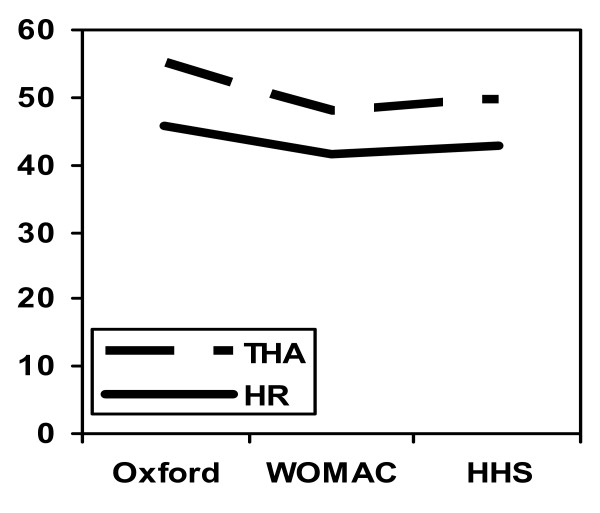
**Improvement in hip scores within our patient cohorts**.

While the patients in each cohort were similar in terms of age and BMI, the preoperative scores in the THA group were worse than those in the HR group. This may be explained by the presence of more advanced disease in the THA group with greater pain and functional disability. This might have made them unsuitable candidates for hip resurfacing procedures. It might also explain why they seemed to have a more significant improvement postoperatively (Figure 4). Patients in both groups displayed excellent functional outcomes with no significant difference between procedures. This was reflected by all scoring tools used.

In terms of gender there was a 1.27:1 ratio of males to females in the THA group compared to a 2.02:1 ratio in the HR group. This may reflect an initial reluctance to use hip resurfacing in females over 50 years old or in those who had less favourable anatomy including dysplasia which is more frequently found in female patients.

Three out of 281 patients (1.1%) of the patients in this study died of causes unrelated to their surgery. While this seems high in a population whose mean age is 57.3 years old, it is within previously described mortality ranges of 4.9% to 15.7% [27,28]. Of the patients who died in both groups, none had revisions up to the time of death.

Both groups in our study had a large proportion of patients who went on to perform high-demand activities such as rowing, skiing, racket sports and running. There was no radiological evidence of wear or its sequelae in either cohort at the time of last follow up. HR offers the young and active patient a femoral bone conserving alternative to THA and may be viewed as a step below THA on the treatment ladder due to the relative ease of conversion if failure occurs. Although the scoring systems we used have been validated it may be that they were not sensitive enough to detect a difference between two different but effective treatment options.

The results of our study suggest that the functional outcome of HR is not superior to custom uncemented THA in the short term and should therefore not be used as the sole basis for deciding which of the procedures to undertake in individual patients. It may be that the potential ease of revision and femoral bone conservation in this group is a driver for the choice of implant especially if both treatments are effective with a high degree of patient satisfaction. It must be remembered that this is a premium cohort of patients ie young and active and highly motivated and in this population any procedure performed well will do well in the short term. In fact until results of revision of HR to THA are known then one needs to be careful in recommending a treatment option which might have a higher early failure rate (United Kingdom National Joint Registry 2007). This might mean that patient selection is more critical for HR than THA.

## Limitations

We recognise that this study has several limitations. These include the lack of randomisation of the patients and a short follow-up period.

## Competing interests

The authors declare that they have no competing interests.

## Authors' contributions

NS collected, tabulated and organised the data. NS and JS wrote the main body of the paper. JS also played a large part as a reviewer and editor. SMA and JH identified the topic as an area of interest, provided the raw data, reviewed, edited and contributed to writing the discussion. We confirm that all authors have read and approved the final manuscript.

## Appendix 1

Modified University of California Los Angeles(UCLA) Activity Scale

1 Inactive: Wholly inactive. Dependent on others. Cannot leave residence.

2 Mostly inactive: Restricted to minimum activities of daily living.

3 Mild activity: Sometimes participates in mild activities such as walking, limited housework and shopping.

4 Regularly participates in mild activities. *Sedentary occupational work*.

5 Moderate activity: Sometimes in moderate activities such as swimming and can do unlimited housework or shopping.

6 Regularly participates in moderate activities. *Light occupational work*.

7 Active Regularly participates in active events such as bicycling, *aqua-aerobics. Gardening or working out in the gym once or twice a week*.

8 Very active: Regularly participates in very active events such as bowling, golf. *Riding, hunting, aerobics. Gardening or working out in the gym three times per week or more. Moderately heavy occupational work. Farming*.

9 Impact sports: Sometimes participates in impact sports such as *running, jogging, tennis, cricket, baseball, rugby, football, hockey, racquet sports, judo, karate and other martial arts*, skiing, acrobatics, ballet dancing, backpacking and *mountaineering. Heavy occupational work*.

10 Regularly participates in impact sports as described above
